# Meta-Analysis: Prognostic Value of Survivin in Patients with Hepatocellular Carcinoma

**DOI:** 10.1371/journal.pone.0083350

**Published:** 2013-12-26

**Authors:** Jin Long Liu, Xue Jun Zhang, Zhao Zhang, An Hong Zhang, Wei Wang, Jia Hong Dong

**Affiliations:** 1 Department of Hepatobiliary Surgery, The Chinese PLA General Hospital, Beijing, China; 2 Department of General Surgery 1, The Affiliated Hospital of Chengde Medical College, Chengde City, China; 3 Department of Vascular Surgery, The People’s Liberation Army 252 Hospital, Baoding City, China; Xiangya Hospital of Central South University, China

## Abstract

**Background:**

The expression of survivin is a promising prognostic indicator for some carcinomas. However, evidence for the prognostic value of survivin with respect to survival in hepatocellular carcinoma remains controversial.

**Aim:**

To conduct a systematic review of studies evaluating survivin expression in hepatocellular carcinoma as a prognostic indicator.

**Methods:**

The relevant literature was searched using PubMed, EMBASE, and Chinese biomedicine databases, and two meta-analyses were performed. One studied the association between survivin expression and the overall survival of patients with hepatocellular carcinoma, whereas the other studied the association between survivin expression and disease-free survival. Studies were pooled, and summary hazard ratios (HRs) were calculated. Subgroup analyses were also conducted.

**Results:**

Fourteen eligible studies with a total of 890 patients were included in this study. Two meta-analyses were performed according to the different outcomes by which prognosis was valued. The combined HR of the overall survival studies was 2.33 (95% CI: 1.65–3.31). The combined HR of disease-free survival studies was 2.13 (95% CI: 1.65–2.75). These data appeared to be significant when stratified by detection method, the language of publication, and HR estimate. The heterogeneities were highly significant (I^2^>50%) when subgroup analyses of overall survival rate were conducted, whereas little heterogeneity was found when subgroup analyses of disease-free survival rate were carried out. The positive expression of survivin in the cytoplasm was significantly correlated with poor prognosis in HCC (HR>1).

**Conclusions:**

This study showed that survivin expression was correlated with poor prognosis in patients with hepatocellular carcinoma, regardless whether they were assessed by overall survival or disease-free survival.

## Introduction

Hepatocellular carcinoma (HCC) is the most common carcinoma in Asia and the fifth most common worldwide, accounting for 1 million deaths every year [1]. The outcome of HCC patients depends predominantly on early diagnosis and a radical cure by surgical treatment [2], [3]. Hepatic resection is one of choices for the treatment of HCC, but there is a significant post-resectional tumor recurrence rate. The post-resectional prognosis of HCC depends mainly on the tumor stage and its biological behavior [4]–[6], which permits only crude stratification of the clinical outcomes for patients with HCC. Therefore, it is important to identify a cellular marker that can predict prognosis. The discovery of molecular prognostic factors may aid the accurate prediction of clinical outcome, and may also reveal novel predictive factors and potential therapeutic targets [7]. Several studies have evaluated prognostic markers that are associated with the clinical outcome of HCC, typically overall or recurrence-free survival. Of these survivin, which is considered an important prognostic marker, has been widely investigated.

Survivin is also known as baculoviral inhibitor of apoptosis repeat containing 5 (BIRC5). It is a member of the inhibitor of apoptosis (IAP) family. It is one of the most cancer-specific proteins identified to date, with unregulated expression in almost all human tumors. Survivin is also highly expressed in fetal tissue, but is undetectable in most terminally differentiated cells [8]. Biologically, survivin inhibits apoptosis, enhances proliferation, and promotes angiogenesis [9]–[11]. The molecular basis for the cancer-specific overexpression of survivin has yet to be fully elucidated. Different studies have suggested that it may originate from the amplification of the survivin locus [12], demethylation of the survivin promoter and exons [13], and increased promoter activity [14] mediated by a variety of oncogenic pathways. Because of the difference in expression between normal and malignant tissue and its causal role in cancer development, survivin is attracting considerable attention as a prognostic indicator in cancer.

The expression of survivin is a promising prognostic indicator for of kinds of carcinomas. Most reports associated it with a worse overall survival of various tumors such as gastric, colorectal, breast, lung, and esophageal cancers. Some of these reports have been confirmed by systematic reviews using meta-analyses [15]–[17]. However, some studies have associated survivin with improved survival such as in pancreatic ductal adenocarcinoma, which was confirmed by a systematic review [18]. However, the prognostic value of survivin for survival in HCC remains controversial. Therefore, we performed a systematic review of the literature followed by meta-analysis.

## Methods

### Literature Search

Studies were identified using electronic searches of PubMed, EMBASE, and Chinese biomedicine databases using the following keywords: [hepatocellular carcinoma] and [BIRC5] or [baculoviral inhibitor of apoptosis repeat-containing 5] or [survivin]. The search ended on April 4, 2013. The references within the identified articles and reviews were then manually searched for additional studies. Finally, we also hand-searched the journals that published articles most relevant to this review.

### Inclusion and Exclusion Criteria

This systematic review generated complete databases from published studies assessing the prognostic value of survivin in patients with HCC. We placed no restrictions on the language of publication. To be eligible for inclusion, studies had to meet the following criteria: (1) they measured survivin expression in HCC using methods such as immunohistochemistry (IHC), polymerase chain reaction (PCR), reverse transcription-polymerase chain reaction (RT-PCR), or western blotting; (2) they correlated overall or disease-free survival with different expression levels of survivin in HCC; (3) they contained a hazard ratio (HR) and 95% confidence interval (CI) for survival according to survivin status, which either were reported or could be calculated from the data in the manuscript; (4) the prognostic effects of survivin were assessed by mortality or the recurrence rate of the patients; (5) when the same author or group reported results from the same patient population in more than one article, the most recent or informative report was included; and (6) the study quality was evaluated >5 stars according to Newcastle-Ottawa quality assessment scale [19].

The exclusion criteria was as follows: (1) letters, reviews, case reports, conference abstracts, editorials, and expert opinion were excluded; and (2) articles in which no information on survival was given, or where HR for survival could not be calculated from the given information.

### Data Extraction

Two investigators (Liu J. L. and Zhang X. J.) reviewed all studies that met the inclusion and exclusion criteria. Data were extracted independently using a data extraction sheet by two investigators (Liu J. L. and Zhang X. J.). The extracted data included the first author’s name, the year of publication, the source of patients, the language of the publication, the number of patients, the type of samples, the assay method, the location of expression, the outcome by which prognosis was valued (overall survival or disease-free survival), and survival data. In addition, controversial problems were resolved in a meeting called by Dong J. H.

### Assessment of Study Quality

Two investigators (Liu J. L. and Zhang X. J.) independently assessed the quality of all studies by reading and evaluating based on the Newcastle-Ottawa quality assessment scale. Briefly, the overall star system assesses three main categories of (1) selection of the cohort, (2) comparability of the cohort, and (3) ascertainment of outcome. A study can be awarded a maximum of one star for each numbered item within the selection and outcome categories, and a maximum of two stars for comparability. The total number of stars was accumulated, with more stars reflecting a higher methodological quality. A study could be awarded a maximum of nine stars.

### Statistical Analysis

Two meta-analyses were performed according to the different outcomes by which prognosis was valued. The primary outcomes of the two meta-analyses revealed the prognostic value of overall survival and disease-free survival in their respective populations, and the outcomes were then stratified by assay method, language of publication, and HR estimate.

HR and 95% CI were used to estimate the effect of survivin expression on survival. A combined HR>1 implied a worse survival for the group with high levels of survivin expression. This negative impact of survivin on survival was considered to be statistically significant if 95% CI for the combined HR did not overlap with 1. If a direct report of HR and 95% CI was not available, an estimated value was derived indirectly from Kaplan-Meier curves using the methods described by Tierney [20]. Kaplan-Meier curves were read using Engauge Digitizer version 4.1 (http://digitizer.sourceforge.net/), and then the survival data read from Kaplan-Meier curves were entered in the spreadsheet based on Tierney et al. [20]. Two independent persons performed this work to reduce inaccuracy in the extracted survival rates.

We used the Cochran Q and I^2^ statistics to assess heterogeneity between studies. For the Q statistic, a *P* value <0.10 was considered to be statistically significant for heterogeneity [21]. The random effects model was then calculated according to the DerSimonian-Laird method [22]. Otherwise, the fixed-effects model (Mantel-Haenszel method) was used. For I^2^, a value >50% was considered to be a measure of severe heterogeneity [23]. All statistical analyses except for Egger’s test were performed using Review Manager 5.0 (http://www.cochrane.org), and Egger’s test was carried out using Stata 12.0. A significant two-way *P* value for comparison was defined as *P*<0.05.

## Results

### Literature Selection

**Figure 1:**
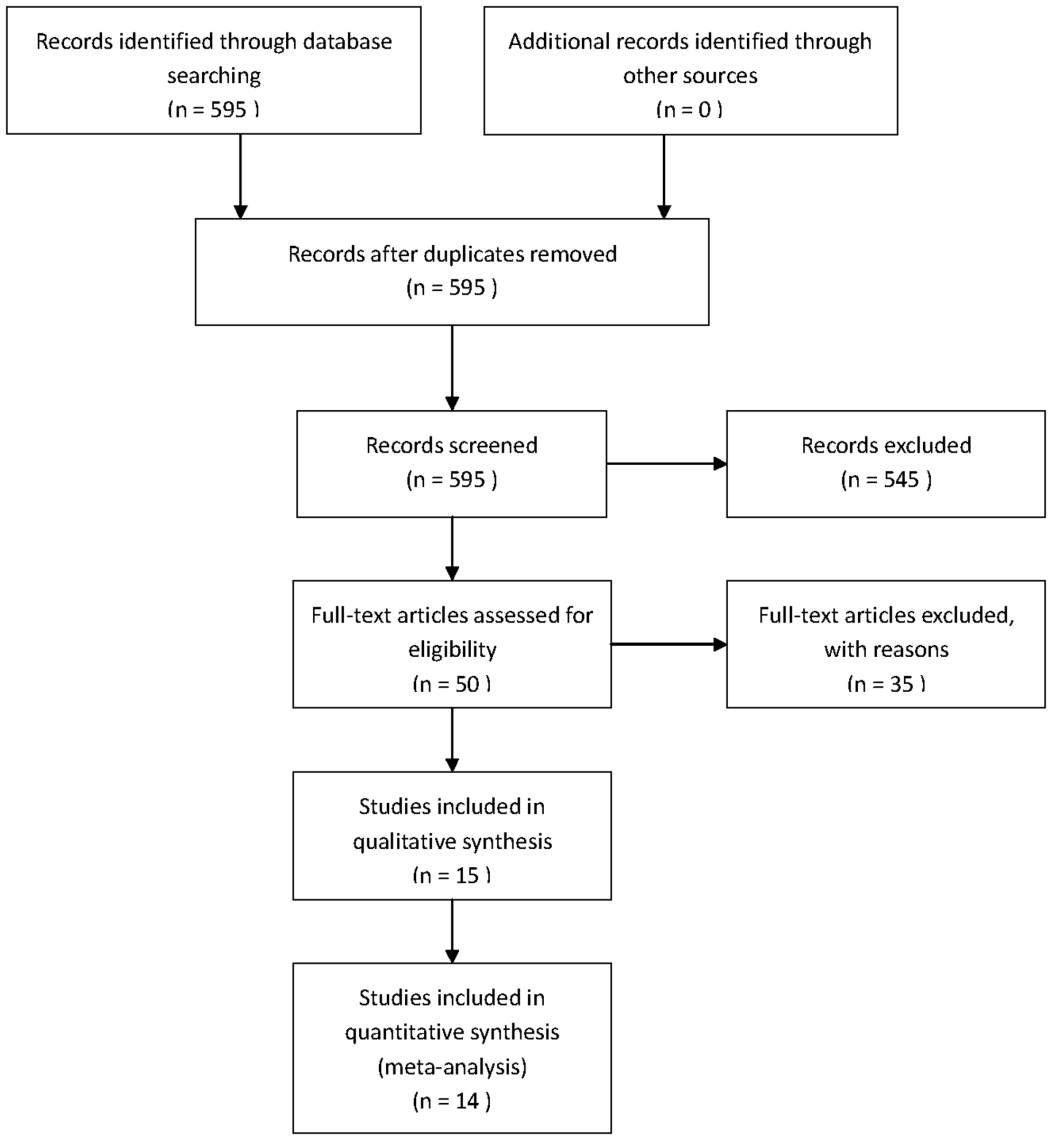
Flow diagram of study selection.

A total of 595 potentially relevant citations were retrieved after the initial database searches. Although an additional 54 studies were found from the references of articles and reviews or by hand-searches of the journals, these were all duplicates of studies from the database searches. Two authors (Liu J. L. and Zhang X. J.) independently read the title and abstract of the relevant articles. Five hundred forty-five citations were excluded from the analysis after the initial screening based on the abstracts or titles, leaving 50 studies available for full-text review. After carefully reading the full-text articles, an additional 35 studies were excluded. Of these 35, 31 studies were excluded because they were reviews or studies using correlation with clinicopathological variables, and not survival. Two studies (performed by the same authors) were excluded due to insufficient survival data [24], [25]. In the three studies that were performed by the same authors, two [26], [27] were excluded and the most informative one [28] was included. As a result, 15 eligible studies [28]–[42] were included in the qualitative analyses and, after the exclusion of one further study [36] due to significant heterogeneity, a total of 890 patients from 14 eligible studies were included in two final meta-analyses (Figure 1).

### Characteristics of the Included Studies

The basic feature descriptions of the 15 studies are summarized in Table 1. Briefly, the study sample sizes ranged from 27 to 94. All of the studies were conducted in Asian populations, except for one [38] that was conducted in the United States. All of the studies investigated survivin expression using hepatocellular carcinoma tissues. Eleven studies [28], [29], [31]–[34], [36]–[38], [41], [42] investigated survivin levels using IHC, two [39], [40] used RT-PCR, one [35] by PCR, and one [30] by western blotting. The cutoff values defining survivin expression were different. Four studies [43]–[46] used the percentage of positive staining rates, whereas six studies [32], [47]–[51] used the sum scores of color intensity and percentage of positive staining as the cutoff values. Four studies [35], [52]–[54] defined the cutoff values by comparing with control samples. One study [55] used the mean value as the cutoff value. Seven studies [28], [31]–[34], [40], [41] valued the prognostic effect by patient mortality, six [29], [30], [35], [37]–[39] by recurrence rate, and two [36], [42] by both mortality and recurrence rate. Eleven studies [28]–[33], [37]–[39], [41], [42] reported survivin as an indicator of poor prognosis, and two [35], [40] showed no significant effect on overall survival. One study [36] reported that survivin is an indicator of good prognosis. In addition, one study [34] correlated nuclear staining of survivin with poor prognosis, whereas cytoplasmic staining showed no significant effect.

**Table 1:** Characteristics and results of included studies.

First Author	Year	NOS	Source	Language	N. of P.	Method	Location	CutoffV.	Outcome	HR Estimate	HR	95% CIs
Ding, W. [28]	2010	8	China	English	70	IHC	Cyt	3 score	Mor.	Sur. Curve	2.02	1.05–3.91
Jiang,C.Y. [31]	2010	7	China	Chinese	81	IHC	Cyt	1 score	Mor.	HR	2.54	1.35–4.80
Lin,H. [32]	2010	9	China	Chinese	76	IHC	Both	1 score	Mor.	HR	1.70	1.02–3.57
Zhang,J. [33]	2008	6	China	Chinese	27	IHC	NA	10%	Mor.	Sur. Curve	5.79	2.26–14.84
Morinaga, S.[40]	2004	8	Japan	English	40	RT-PCR		Meanvalue	Mor.	HR	0.73	0.05–11.93
Yang, Y. [41]	2011	7	China	English	63	IHC	Cyt	3 score	Mor.	HR	7.97	2.81–22.62
Zhang,Y.R. [34]	2007	7	China	Chinese	54	IHC	Cyt	10%	Mor.	Sur. Curve	1.24	0.71–2.17
					52	IHC	Nu	10%	Mor.	Sur. Curve	2.71	1.21–6.08
Ye,C.P. [42]	2007	7	China	English	55	IHC	Cyt	1 score	Mor.	Sur. Curve	2.03	1.14–3.60
								1 score	Rec.R	Sur. Curve	2.12	1.18–3.82
Chau, G. Y. [36]	2007	8	Taiwan	English	94	IHC	Cyt	Control	Mor.	Sur. Curve	0.48	0.29–0.77
								Control	Rec.R	Sur. Curve	0.60	0.38–0.97
Hui,W.T. [29]	2008	6	China	English	42	IHC	Cyt	10%	Rec.R	Sur. Curve	2.01	1.01–4.00
Guo,R.H. [30]	2011	9	China	Chinese	52	W Blot		Control	Rec.R	HR	8.53	1.97–36.96
Zhu,W. [35]	2010	9	China	Chinese	82	PCR		Control	Rec.R	Sur. Curve	1.56	0.88–2.79
Fields, A. C. [38]	2005	8	America	English	72	IHC	Nu	10%	Rec.R	Sur. Curve	2.35	1.26–4.36
Ikeguchi, M. [39]	2002	9	Japan	English	51	RT–PCR		Control	Rec.R	HR	2.52	1.21–5.26
Cho, S. [37]	2010	7	Korea	English	73	IHC	Both	5 score	Rec.R	Sur. Curve	2.05	1.09–3.87

NOS, Newcastle-Ottawa quality assessment scale; N.of P.,number of patients; Cutoff of V.,cutoff of value; immunohistochemistry RT-PCR, reverse transcription polymerase chain reaction; W Blot, western blot;Cyt.,Cytoplasm; Nu.,nucleus; Mor.,mortality; Rec.R,reccurence rate; Sur. Curve, survival curve; NA, not applicable.

### Methodological Quality of the Studies

To assess the quality of the included studies, two authors independently extracted data and assessed the methodological quality using the Newcastle-Ottawa quality assessment scale. The scores are shown in Table 1. The studies included in our meta-analysis all had high levels of methodological quality (>5 stars on the Newcastle-Ottawa scale).

### Assessment of Heterogeneity

**Figure 2:**
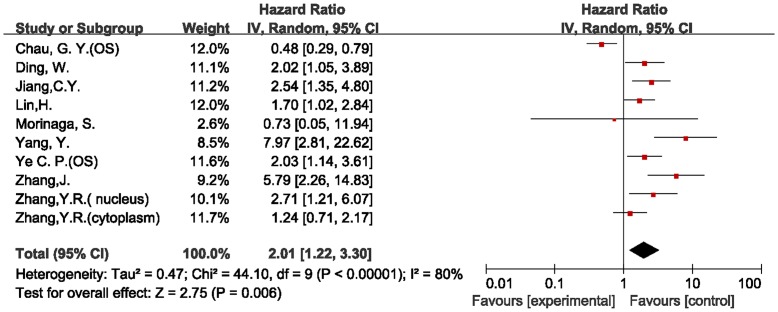
Forest plot of Hazard ratio (HR) for overall survival (OS) of HCC patients. Highly significant heterogeneity can be found before Chau GY’ study was excluded.

**Figure 3:**
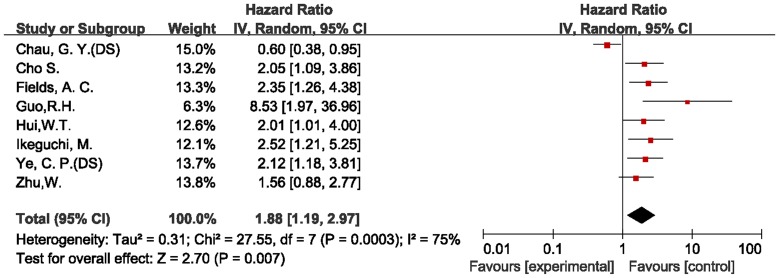
Forest plot of Hazard ratio (HR) for disease-free survival (DS) of HCC patients. Highly significant heterogeneity can be found before Chau GY’ study was excluded.

When all of the 15 eligible studies were pooled according to the different outcomes of patients, both of the combined HR showed that the expression of survivin had an inverse effect on survival in HCC. However, we detected highly significant heterogeneity in both of the final meta-analyses of overall survival (chi-squared = 44.10, I^2^ = 80%, *p*<0.00001) and disease-free survival (chi-squared = 27.55, I^2^ = 75%, *p = *0.0003). We then easily identified the source of the heterogeneities from both of the forest plots (Figures 2, 3). The heterogeneities in the two meta-analyses were from the same study [36], which examined the association of survivin expression with both overall and disease-free survival in patients with HCC.

**Figure 4:**
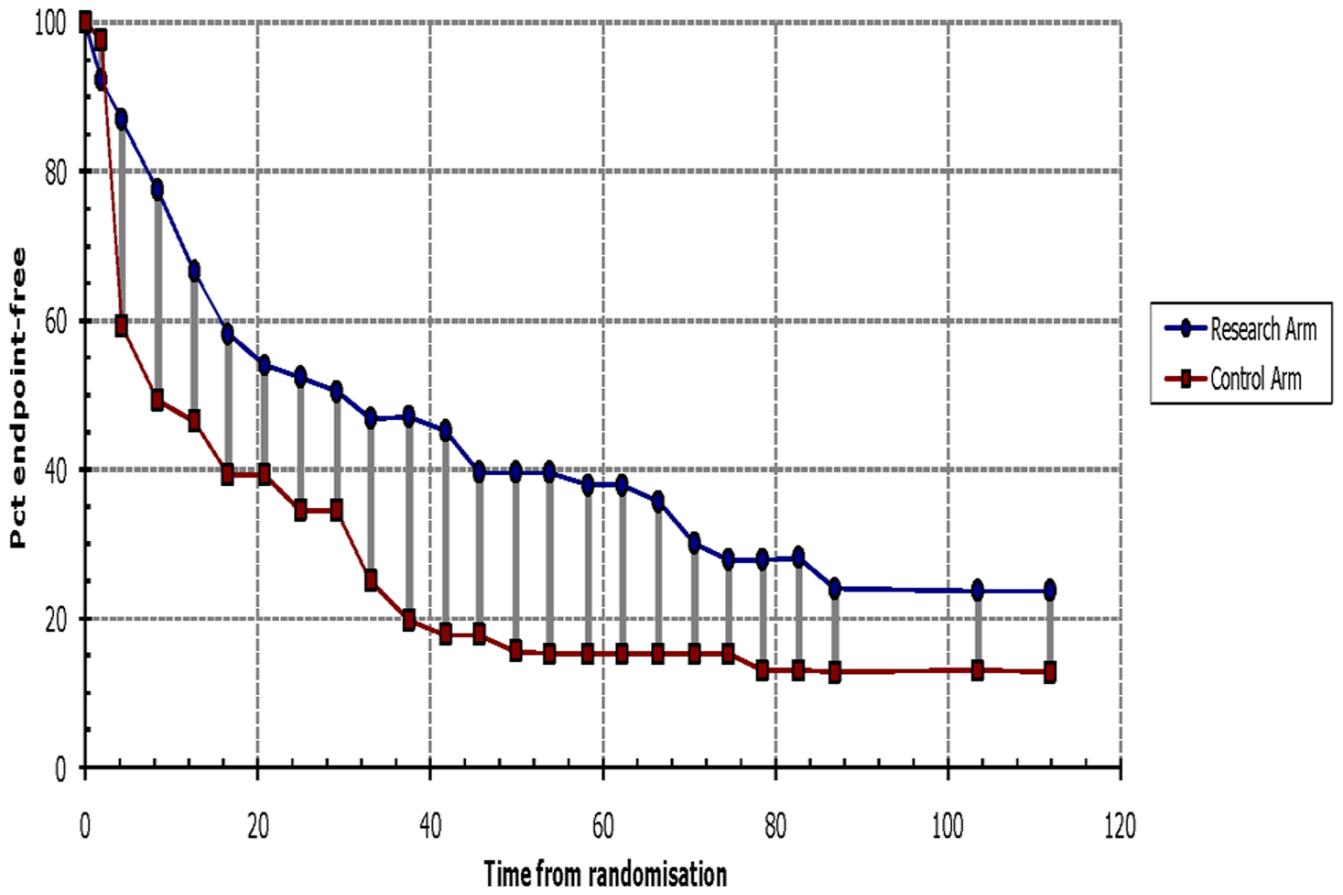
Survival data for overall survival based on Kaplan-Meier curve by Tierney’s method from Chau GY’ study. The survival data read from Kaplan-Meier curves by Engauge Digitizer version 4.1were entered in the spreadsheet appended to Tierney’s paper, then we got this figure and HR value. It is similar to the orignal graph, see figure S1.

**Figure 5:**
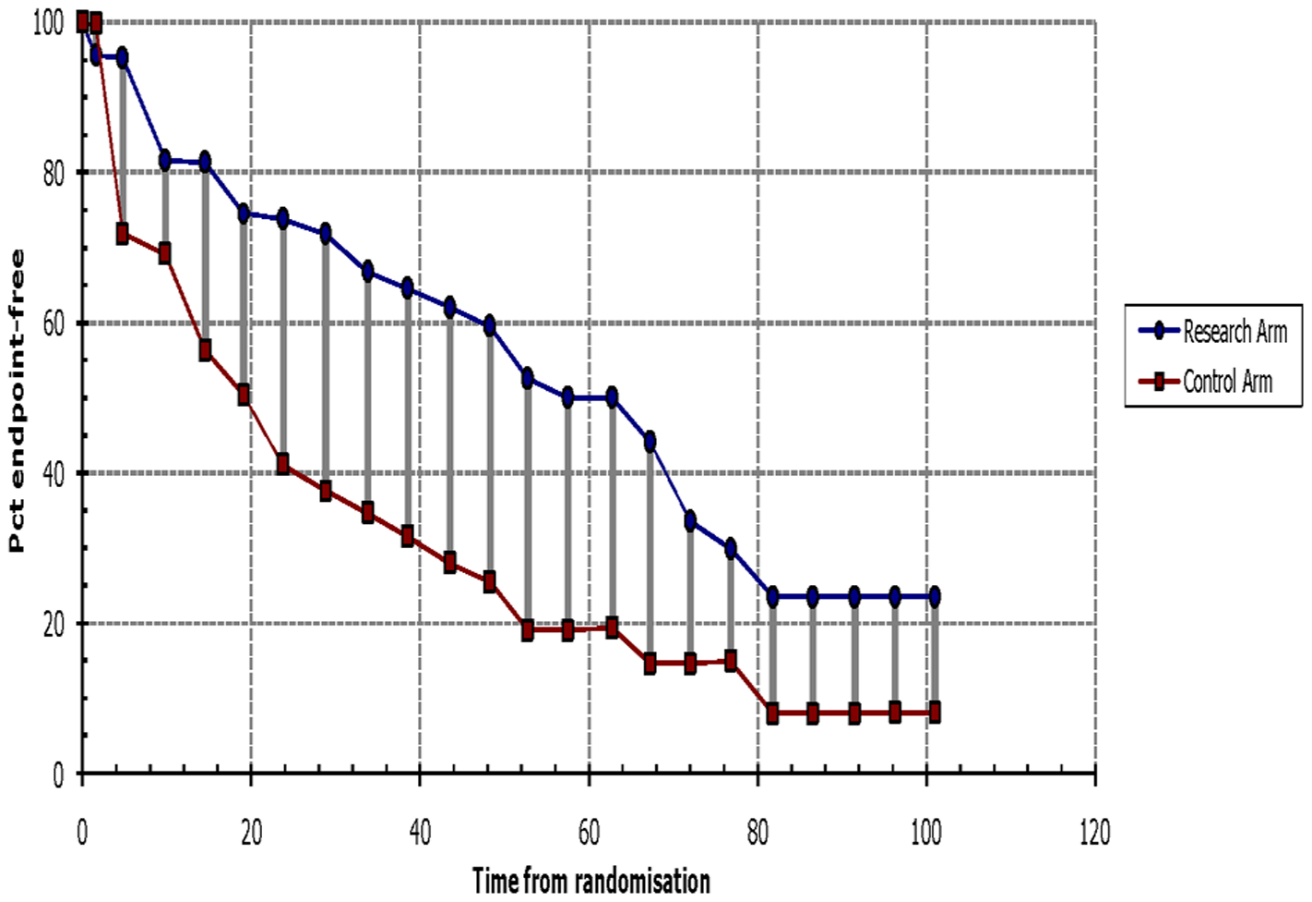
Survival data for disease-free survival based on Kaplan-Meier curve by Tierney’s method from Chau GY’ study. It is similar to the orignal graph(figure S2),which means the HR value we extrated is close to the original value.

In a previous study [36], the Kaplan-Meier method was used to assess cytoplasmic survivin expression, which was reported to be an indicator of good prognosis (Figure S1 and S2). However, multivariate analysis suggested that survivin expression did not correlate with disease-free or overall survival. The authors did not provide the HR value from the multivariate analysis, and so calculated the HR from the Kaplan-Meier curve using Tierney’s method (Figures 4 and 5). This is the only study that gave inconsistent results by different methods of analysis, and these inconsistent results may be the source of the heterogeneity. For this reason, we excluded this study from meta-analysis. The heterogeneities then decreased, and the final conclusions of the meta-analysis were unaffected (Tables 2 and 3).

**Table 2:** Summarized HRs and subgroup analyses for survivin on HCC of overall survival.

	N. of studies	Number of patients	HR(95%CIs)	Heterogeneity test		
				chi-squared	I^2^	P-value
Overall	8	518	2.33 (1.65–3.31)	16.08	50%	0.04
Methods						
IHC	7	478	2.38 (1.67–3.41)	15.51	55%	0.03
Language						
English	4	228	2.63 (1.35–5.10)	6.36	53%	0.1
Chinese	4	290	2.21 (1.41–3.47)	9.20	57%	0.06
HR Estimatie						
HR	4	260	2.65 (1.32–5.32)	7.50	60%	0.06
Sur.Curve	4	258	2.19 (1.41–3.40)	8.26	52%	0.08
Location						
Cytoplasm	5	323	2.26(1.41–3.62)	10.06	60%	0.04

**Table 3:** Summarized HRs and subgroup analyses for survivin on HCC of disease-free survival.

	N. of studies	Number of patients	HR(95%CIs)	Heterogeneity test		
				chi-squared	I^2^	P-value
Overall	7	427	2.13 (1.65–2.75)	4.92	0%	0.55
Methods						
IHC	4	242	2.13 (1.56–2.92)	0.14	0%	0.99
Language						
English	5	293	2.19 (1.64–2.92)	0.30	0%	0.99
Chinese	2	134	3.17 (0.61–16.40)	4.48	78%	0.03
HR Estimatie						
HR	2	103	3.22 (1.67–6.21)	2.13	53%	0.14
Sur.Curve	5	324	1.98 (1.51–2.61)	1.02	0%	0.91
Location						
Cytoplasm	2	97	2.07(1.33–3.24)	0.01	0%	0.91

A total of 890 patients from the 14 included studies were distributed to the two meta-analyses according to patient outcome. Heterogeneity disappeared in the final meta-analysis of disease-free survival (chi-squared = 4.92, I^2^ = 0%, *p = *0.55). Although heterogeneity still occurred in the final meta-analysis of overall survival, it dropped to the level where random effects meta-analyses could be conducted without great error (chi-squared = 16.08, I^2^ = 50%, *p = *0.04).

### Results of Meta-analysis

We performed two final meta-analyses according to the different outcomes of HCC patients. The results are shown in Tables 2 (overall survival) and 3 (disease-free survival). The combined HR of the overall survival studies was 2.33 (95% CI: 1.65–3.31). In the subgroup analysis according to the method of survivin detection used, the combined HR was 2.38 (95% CI: 1.67–3.41) for IHC after excluding one study that used RT-PCR [40], but the heterogeneity was highly significant (chi-squared = 15.51, I^2^ = 55%, *p* = 0.03). When stratified according to publication language, the combined HR of both the English (HR = 2.63, 95% CI: 1.35–5.10) and Chinese (HR = 2.21, 95% CI: 1.41–3.47) literature showed an inverse effect on survival, and the heterogeneities were both highly significant (I^2^>50%). When the HRs that were extracted directly from the four evaluable studies were pooled, the combined HR was 2.65 (95% CI: 1.32–5.32). When the HRs calculated indirectly from Kaplan-Meier curves were pooled, the combined HR was 2.19 (95% CI: 1.41–3.40). The heterogeneities were also both highly significant (I^2^>50%). The combined HRs indicated that survivin expression was associated with poor prognosis in patients with HCC when measured by overall survival rate. However, the heterogeneities were highly significant, and so the data should be considered with caution. To further investigate the relationship between the subcellular localization of survivin and overall survival, five studies [45], [48]–[51] that reported cytoplasmically localized survivin in 323 patients were included in the meta-analysis. The combined HR was 2.26 (95% CI: 1.41–3.62), which demonstrated that the positive expression of survivin in the cytoplasm was significantly correlated with poor prognosis in HCC.

The combined HR of the disease-free survival studies was 2.13 (95% CI: 1.65–2.75). In the subgroup analysis according to the method of survivin detection used, the combined HR was 2.13 (95% CI: 1.56–2.92) for IHC after the exclusion of one study [39] for RT-PCR, one [35] for PCR, and one [30] for western blotting. There was no heterogeneity (I^2^ = 0%). When studies were stratified according to the publication language, the combined HR of English language publications showed an inverse effect on survival (HR = 2.19, 95% CI: 1.64–2.92) without heterogeneity (I^2^ = 0%). The non-English literature publications demonstrated highly significant heterogeneity (I^2^ = 78%), and did not show any effect on disease-free survival (HR = 3.17, 95% CI: 0.61–16.40). When the HRs that were extracted directly from the two evaluable studies [30], [39] were pooled, the combined HR was 3.22 (95% CI: 1.67–6.21), and the heterogeneity was highly significant (I^2^ = 53%). When the HRs calculated indirectly from the Kaplan-Meier-based survival curves were pooled, there was no heterogeneity (I^2^ = 0%), and the combined HR was 1.98 (95% CI: 1.51–2.61). The combined HR from the subgroup indicated that survivin expression was associated with poor prognosis in patients with HCC when measured by disease-free survival. The relationship between the subcellular localization of survivin location and disease-free survival was also assessed. Two studies [43], [50] that reported cytoplasmic survivin in 97 patients were included in the meta-analysis. The combined HR was 2.07 (95% CI: 1.33–3.24), which demonstrated that the positive expression of survivin in the cytoplasm was significantly associated with HCC recurrence.

### Publication Bias

**Figure 6:**
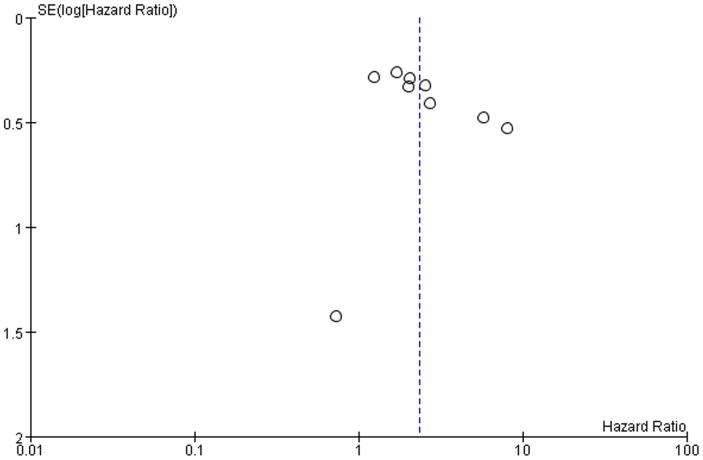
Funnel plots were used to detect publication bias on overall estimate of overall survival. Studies are distributed symmetrically, and suggest that publication bias is absence in the meta-analysis.

**Figure 7:**
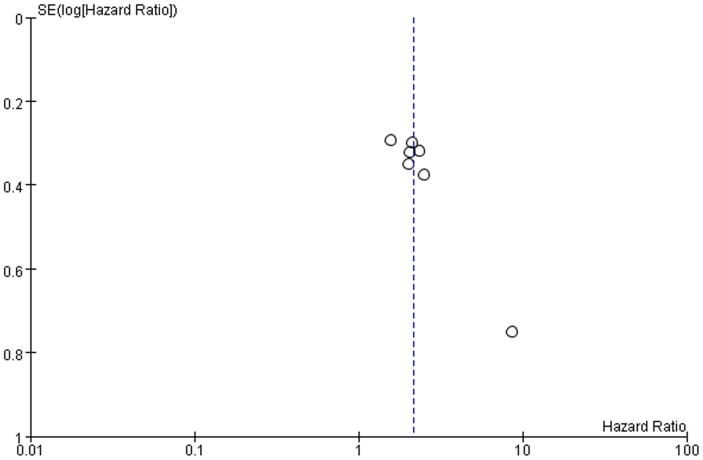
Funnel plots were used to detect publication bias on overall estimate of disease-free survival. Studies are distributed symmetrically, and suggest that publication bias is absence in the meta-analysis.

Publication bias may exist when non−significant findings remain unpublished, which can artificially inflate the apparent magnitude of an effect. Funnel plots of the two meta-analyses are shown in Figures 6 and 7. There was no obvious funnel plot asymmetry in any of the included studies. We also perform Egger’s test using Stata 12.0, and the *p*-values were both greater than 0.1 (overall survival *p* = 0.31, disease-free survival, *p* = 0.18). Therefore, there was no evidence of publication bias.

## Discussion

The potential of survivin as a biomarker for the prognosis of different malignancies has generated significant interest. However, the conclusions of published studies investigating its prognostic value for different cancers are contradictory. Survivin expression is an unfavorable prognostic indicator for esophageal and lung cancers [15], [16]. In contrast, a positive prognosis associated with nuclear survivin expression has been reported for pancreatic ductal adenocarcinoma [18]. Many studies have investigated the prognostic value of survivin in HCC, but the sample size of all these studies have been small. In addition, reports of the prognostic significance of survivin in HCC are controversial. No meta-analyses or review protocols have been reported previously for the prognostic value of survivin in HCC. We performed a systematic review to evaluate the role of survivin for the prognosis of HCC using a larger sample size.

In this review, 15 studies were included based on the inclusion and exclusion criteria. One study [36] was then excluded from the meta-analysis as a source of highly significant heterogeneity. This study examined the association of survivin expression with both overall and disease-free survival in patients with HCC. It reported that the expression of survivin was an indicator of good prognosis by univariate analysis. However by multivariate analysis, survivin did not correlate with disease-free or overall survival. Although different outcomes were obtained by different analyses, the HR value from multivariate analysis was more reasonable. However, we were unable to acquire it from the original article, and so this study was excluded from the final meta-analysis. Our conclusions from both meta-analyses were not altered after excluding this study, indicating that our conclusions are stable and convincing.

Ultimately, we enrolled 14 studies that correlated the expression of survivin the overall survival and disease-free survival of HCC patients for meta-analysis. In the included studies, survivin expression was detected using IHC, RT-PCR, PCR, or western blotting. All specimens were hepatocellular carcinoma tissues. Using meta-analyses of the 14 studies, we identified that survivin expression was associated with poor prognosis in hepatocellular carcinoma. This observation can be explained by the ability of survivin to inhibit apoptosis, promote proliferation, and increase angiogenesis. Because of these functions, survivin is likely to be causally involved in tumor progression and, so increased levels would be expected to predict a poor prognosis.

The subcellular distribution of survivin appears to alter during progression through the cell cycle. For example, survivin was associated with the microtubule organization center during interphase and centrosomes and mitotic spindles at metaphase, but was relocated to midbodies during late telophase [56], [57]. To investigate the relationship between the subcellular localization of survivin and the prognosis of HCC, we performed subgroup analyses of studies in which survivin expression was located in the cytoplasm. Data revealed that the cytoplasmic expression of survivin was closely correlated with poor prognosis of HCC patients, regardless of being assessed by overall survival or disease-free survival. However, there is a lack of studies reporting nuclear expression of survivin in each meta-analysis group, and so further work is necessary to establish whether the nuclear expression of survivin is associated with the prognosis of HCC.

Some limitations of this review must be addressed. The most important concern is whether these data can be extrapolated to other races. The HCC patients included in most studies (13/14) were Asian, and so, the results of our study should be compared with other races. Additional studies in HCC patients of other races are needed to further clarify our results. Additional defects of our meta-analysis were problems with heterogeneity, although most of these were not highly significant. Nevertheless, it is possible that the results of this meta-analysis could have been influenced by the heterogeneities. Therefore, we attempted to perform a stratified subgroup analysis according to the characteristics of the patients that could be acquired from the studies. However, some characteristics could not be obtained from the available data. The method used to extrapolate the HR could also be a potential source of bias. If the HR was not reported in a study or it could not be calculated from the data included in the article, we extrapolated it from the survival curves using Tierney’s method. However, extrapolating HR from survival curves seemed to be less reliable than when HR was obtained directly from published statistics. Finally, although the absence of publication bias was identified by Egger’s test, some studies were not included in the meta-analysis due to insufficient survival data. Therefore, it is possible that the outcome of the meta-analysis might be altered if these studies were included. For these reasons, the pooled HRs calculated in our meta-analysis may be overestimated, and our results should be substantiated by additional prospective studies.

## Conclusions

Survivin expression was correlated with poor prognosis in patients with HCC in this systematic review with meta-analysis, regardless of being valued by overall survival or disease-free survival. As a prognostic factor for HCC, survivin may assist a more accurate prediction of the clinical outcome of HCC, and may also be a novel therapeutic target. Nevertheless, our study has some limitations, and so our conclusions should be confirmed by an adequately designed prospective study. The exact role of survivin expression should also to be determined by an appropriate multivariate analysis that considers the classic well-defined prognostic factors for HCC.

## Supporting Information

Figure S1 **Overall survival curves for survivin high expression versus low expression from Chau GY’ study.**
(TIF)

Figure S2 **Disease-free survival curves for survivin high expression versus low expression from Chau GY’ study.**
(TIF)

Checklist S1 (DOC)
