# Supplementary figures and images for: Meta-Analysis: Prognostic Value of Survivin in Patients with Hepatocellular Carcinoma
Source: PLoS One. 2013 Dec 26;8(12):e83350. doi: 10.1371/journal.pone.0083350 (PMC3873280; doi:10.1371/journal.pone.0083350)

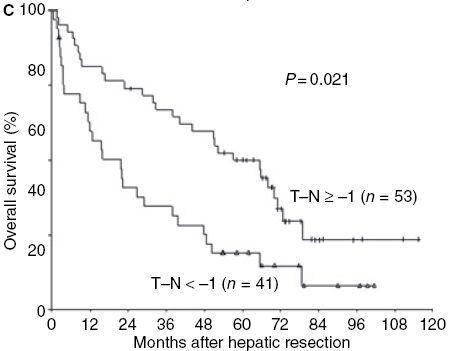

Supplement: Figure S1 — Overall survival curves for survivin high expression versus low expression from Chau GY’ study. (TIF) [file pone.0083350.s001.tif]

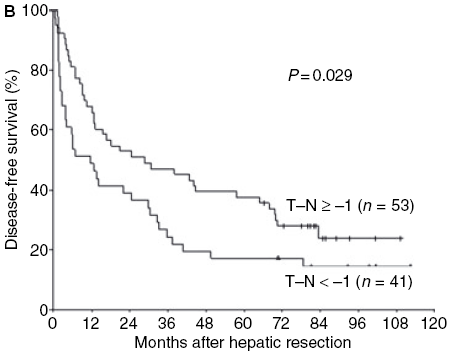

Supplement: Figure S2 — Disease-free survival curves for survivin high expression versus low expression from Chau GY’ study. (TIF) [file pone.0083350.s002.tif]
